# Cutaneous fungal infection in an immunocompromised host

**DOI:** 10.1099/jmmcr.0.005101

**Published:** 2017-06-21

**Authors:** David Sotello, Mark Cappel, Tamara Huff, Diana Meza, Salvador Alvarez, Claudia R. Libertin

**Affiliations:** ^1^​ Division of Infectious Diseases, Mayo Clinic, Jacksonville, FL, USA; ^2^​ Department of Dermatology, Mayo Clinic, Jacksonville, FL, USA; ^3^​ Department of Laboratory Medicine and Pathology, Mayo Clinic, Jacksonville, FL, USA

**Keywords:** hyalohyphomycosis, *Purpureocillium lilacinum*, ascending subcutaneous nodules, voriconazole

## Abbreviations

ITS, internal transcribed spacer; TEF, Translation elongation factor.

## Case summary

**Fig. 1.**
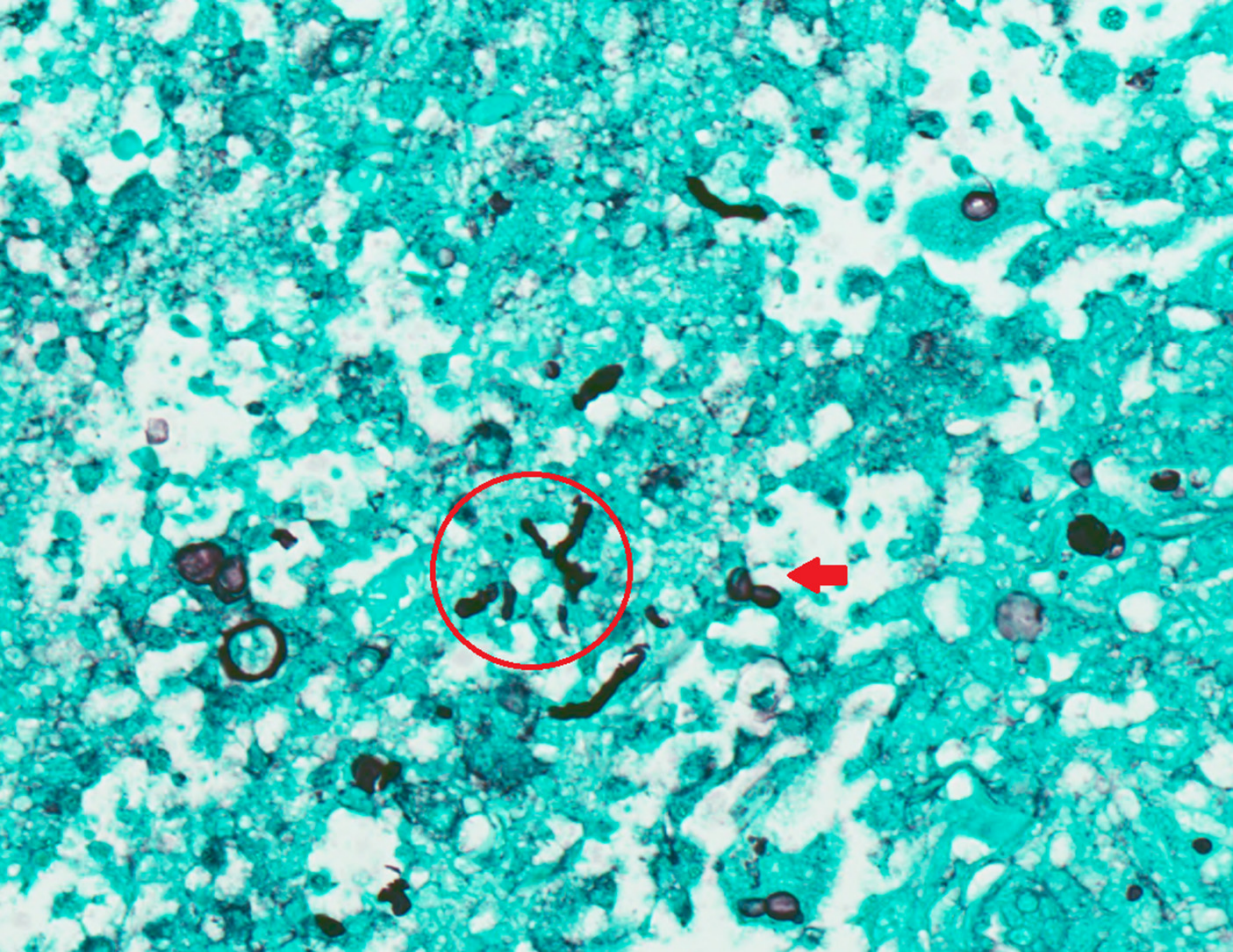
Right arm skin nodule biopsy. Gomori methenamine silver stain (×400), showing broad-based budding yeast (arrow) and with some hyphae (indicated by a circle).

A 69-year-old male, who had a second orthotropic liver transplant for hepatic allograft failure from relapsed primary sclerosing cholangitis of the graft 4 months earlier, presented with nodular lesions of the right arm. He was cytomegalovirus donor positive and recipient negative, Epstein-Barr virus recipient positive and QuantiFERON-TB Gold test negative. He had no recent episodes of acute rejection. He was immunosuppressed on 2 mg tacrolimus twice daily, 500 mg mycophenolate mofetil twice daily and 10 mg prednisone daily. Two months earlier, he noted a tender nodule on the dorsum of the right hand, which progressed to multiple tender nodules tracking up his arm towards his axilla. He denied any systemic symptoms such as fever, chills, weight loss or night sweats. He denied a history of trauma. He spent most of his time inside his house with his dog. He was not a gardener. He resided in the south-eastern region of the USA. A biopsy performed by a local dermatologist demonstrated granulomatous inflammation, and yeast with a broad base and some hyphal forms. No cultures were performed. Physical examination revealed a lesion on the dorsum of the right hand that was verrucous in appearance. The other lesions of the upper extremity were tender, indurated, erythematous nodules. After being evaluated in our infectious and dermatology departments, a repeat biopsy was performed and is shown in Fig. 1.

**Question**

What organism is the highest in your differential to grow in cultures?

**Answer options**

1. *Sporothrix schenckii*

2. *Blastomyces dermatitidis*

3. *Candida parapsilosis*

4. *Histoplasma capsulatum*

5. *Purpureocillium lilacinum*

## Discussion

**Correct Answer:** 5. *Purpureocillium lilacinum*.

The differential diagnosis of skin nodules in an immunocompromised host is broad and includes bacterial, mycobacterial, fungal and other non-infectious aetiologies. In our case, the differential was narrowed by the presence of the fungal hyphal elements seen in the skin biopsy. All of the answer options in the question above are associated with cutaneous lesions. The description of broad-based yeast is very suggestive of *Blastomyces dermatitidis*, but the correct answer is *Purpureocillium lilacinum*. Our case is intended to highlight the differences of mould appearances in histological tissue. The appearance of a mould such as *Purpureocillium lilacinum*, a moniliaceous hyphomycete, is distinctly different than a dimorphic fungus like *B. dermatitidis*. The moniliaceous and hyaline hyphomycetes show hyphal elements in tissue, but *Blastomyces* does not.

*Purpureocillium lilacinum*, a filamentous saprobic fungus, was previously known as *Paecilomyces lilacinus. Purpureocillium lilacinum* is a ubiquitous fungus found in the environment, especially in the soil. It can be found in air and water, as well in fertilizers due to its nematicidal activity [1]. Histological examination usually shows hyphae, phialides and conidia that may sporulate in infected tissues [2]. These hyphal elements were seen in our patient’s histopathology. Hyalohyphomycosis is a fungal infection caused by various moulds, one of which is *Purpureocillium lilacinum*. This is in contrast to *Blastomyces,* which typically is seen as large broad-base unipolar budding yeast cells measuring 8–20 microns in size in human tissue [3]. *B. dermatitidis* is dimorphic; it grows as a mould at room temperature, but as yeast at body temperatures [3]. The presence of hyphal elements in human tissue excludes blastomycosis.

This mould, *Purpureocillium lilacinum*, had a microscopic morphology that showed conidiophores mostly arising from hyphae, bearing branches with phialides and oval conidia in chains. Cultures became positive in all three media used: brain heart infusion with chloramphenicol and gentamicin agar (Remel), Sabouraud brain heart infusion agar (Remel), and inhibitory mold agar (Remel) on which the colonies displayed a lilac colour. Molecular techniques are usually performed for confirmatory species identification, usually through DNA sequencing of different targets, including the internal transcribed spacer (ITS) region and translation elongation factor (TEF) 1-α encoding gene for *Purpureocillium lilacinum* [4]. Matrix-assisted laser desorption ionization–time of flight mass spectrometry has also been used [5]. The species of our isolate was confirmed using DNA sequencing of the ITS and TEF 1-α targets, which was performed by the Fungus Testing Laboratory at the University of Texas Health Science Center at San Antonio (TX, USA). The ITS and TEF 1-α DNA sequences for this isolate (UTHSCSA DI17-39) have been deposited into GenBank (accession numbers MF099428 and MF099429, respectively).

*Purpureocillium lilacinum* has been described as an emerging pathogen both in immunocompetent and immunocompromised patients [2]. This is probably due to increased prevalence of the immunocompromised in the general population and better diagnostic techniques. In immunocompetent patients, *Purpureocillium lilacinum* infection presents as a localized process, usually as skin or ocular infections [2]. *Purpureocillium lilacinum* has been reported in cases of peritonitis associated with peritoneal catheter [6], endovascular infections [7], sinusitis, pulmonary infections and catheter-related fungaemia [8]. It has also been associated with foreign bodies, including prosthetic devices [4, 9], and as a contaminant of antiseptic lotions/solutions. In the immunocompromised population, *Purpureocillium lilacinum* infections have been described to occur in recipients of renal, liver, heart [9] and bone marrow transplants [10], and other debilitating conditions like haematophagocytic syndrome [11], where it can produce localized or invasive disease.

There are no available guidelines for the management of *Purpureocillium* *lilacinum* infections. Appropriate identification is crucial, since the effective therapeutic agents can differ from other fungal species that it can be phenotypically mistaken for, like *Paecilomyces* spp. [5]. As with our patient’s isolate, *Purpureocillium lilacinum* is usually resistant to amphotericin B. Fluconazole and itraconazole usually have limited activity against this species. Voriconazole and posaconazole have been used effectively and are the recommended first-line agents, especially for invasive disease. Terbinafine has been successfully used for cutaneous infection [12]. Duration of antifungal therapy is unclear, but a minimum of 3 months has been described for localized disease. Longer treatments may be required for invasive presentations. Surgical debridement should be considered if amenable due to anatomical location or other factors [1, 9]. Our patient was cured of his infection by receiving 3 months of voriconazole 200 mg by mouth twice a day (with 2 years of follow-up).
